# Changes in the Secretion of Melatonin and Selected Adipokines during the Progression of Parkinson’s Disease—Preliminary Studies

**DOI:** 10.3390/metabo13050668

**Published:** 2023-05-18

**Authors:** Jan Milanowski, Kamil Kozerawski, Weronika Falęcka, Dominik Dudek, Beata Lisewska, Paweł Lisewski, Jarosław Nuszkiewicz, Roland Wesołowski, Jakub Wojtasik, Celestyna Mila-Kierzenkowska, Karolina Szewczyk-Golec

**Affiliations:** 1 Students Research Club of Medical Biology, Department of Medical Biology and Biochemistry, Faculty of Medicine, Ludwik Rydygier Collegium Medicum in Bydgoszcz, Nicolaus Copernicus University in Toruń, 85-092 Bydgoszcz, Poland; 2 Medical Center “Neuromed”, 85-163 Bydgoszcz, Poland; 3 Department of Medical Biology and Biochemistry, Faculty of Medicine, Ludwik Rydygier Collegium Medicum in Bydgoszcz, Nicolaus Copernicus University in Toruń, 85-092 Bydgoszcz, Poland; jnuszkiewicz@cm.umk.pl (J.N.); roland@cm.umk.pl (R.W.); 4 Centre for Statistical Analysis, Nicolaus Copernicus University in Toruń, Chopina 12/18 St., 87-100 Toruń, Poland; jwojtasik@umk.pl

**Keywords:** adiponectin, dyskinesia, leptin, melatonin, Parkinson’s disease, resistin

## Abstract

Parkinson’s disease (PD) is one of the most common neurodegenerative diseases affecting elderly people. Considering the gap in the literature on melatonin and adipokine levels in PD patients at various stages of the disease, we conducted a study to investigate the levels of selected parameters in PD patients at the disease’s early (ES) and advanced (AS) stages. Melatonin, leptin, adiponectin, and resistin concentrations were measured in the blood serum of 20 PD patients without dyskinesia (ES), 24 PD patients with dyskinesia (AS), and 20 healthy volunteers as a control group (CG). The data were analyzed using ANOVA. Melatonin was significantly lower in ES (*p* < 0.05) and higher in AS patients (*p* < 0.05) compared to CG. The level of leptin was increased both in ES (*p* < 0.001) and AS (*p* < 0.001) versus CG, while resistin was increased only in patients with dyskinesia (*p* < 0.05). Higher melatonin (*p* < 0.001) and resistin (*p* < 0.05) and lower leptin (*p* < 0.05) levels were found in AS versus ES. The main findings of the study include the changes in inflammatory markers’ levels during PD and a surprising increase in melatonin level in dyskinesia patients. Further research is necessary, which will be aimed at modulating the secretion of melatonin and adipokines as a treatment target for PD.

## 1. Introduction

Parkinson’s disease (PD) is one of the most common neurodegenerative diseases affecting elderly people. An estimated 6.1 million people had PD diagnosed in 2016 [1]. PD usually occurs in patients over 50 years old and increases in prevalence with age. Its peak is between the ages of 85 and 89 [2]. It is caused by a declining quantity of dopaminergic neurons and α-synuclein accumulation in substantia nigra [3,4]. α-Synuclein contributes to PD pathogenesis in a number of ways. It is generally thought that its aberrant soluble oligomeric conformations (protofibrils) mediate the disruption of cellular homeostasis and neuronal death through the effects on various intracellular targets, including synaptic function. Moreover, α-synuclein secretion may exert deleterious effects on neighboring cells, including aggregation seeding, thus possibly contributing to disease propagation [5]. PD is characterized by motor disorders such as dyskinesia, bradykinesia, or instability. Furthermore, non-motor symptoms (NMS) include constipation, pain, and depression. Some researchers report weight loss in PD patients [6]. Disturbed sleep associated with the disruption of circadian rhythms characterizes the early stage of PD (premotor phase). Additionally, olfactory disorders, pains, and autonomic dysfunctions accompany this stage of the disease. Progression is associated with the deterioration of motor features by dyskinesia. The late stage of the disease is generally treatment-resistant and is characterized by instability, falls, dysphagia, dysfunctional speech, and urinary incontinence [7]. Preclinical and clinical studies have shown that melatonin (N-acetyl-5-methoxytryptamine) supplementation could be an appropriate therapy for PD [8,9,10,11,12]. Administration of melatonin has been found to cause the inhibition of some pathways related to apoptosis, autophagy, oxidative stress, inflammation, α-synuclein aggregation, and dopamine loss in PD. Melatonin is a neurohormone synthesized and secreted from the pineal gland [13]. This hormone plays an essential role in maintaining homeostasis and is a potent antioxidant. Among the mechanisms of its action, the induction of complex antioxidant and DNA repair systems should be mentioned [14]. However, the primary function of melatonin is to regulate circadian rhythms [15]. The patients with PD have been found to have an inverted circadian blood pressure rhythm, which is characterized by postprandial hypotension and nocturnal hypertension, and, moreover, PD patients have shown a decrease in sympathetic activity during a day [16]. This may cause the sympathetic morning peak of melatonin secretion to disappear. Some novel research has discovered that melatonin can greatly improve subjective and objective quality of sleep, and has therefore shown promise in reducing the severity of insomnia among patients with PD [17,18]. According to the paper published by Hu et al. [19], there are currently around eight clinical trials registered on clinicaltrials.gov and the EU Clinical Trials Register which are investigating the therapeutic potential of melatonin and its analogues for treating sleep disorders that are commonly associated with PD.

The secretion and function of several hormones synthesized by the adipose tissue, namely adipokines, have also been linked to PD. Leptin, the first described adipokine, is a polypeptide hormone composed of 146 amino acid residues [20,21]. Numerous leptin receptors are located in different brain parts, such as the hypothalamus, hippocampus, cortex, and olfactory bulb [22]. Leptin, by stimulating various brain areas, contributes to the modulation of cortisol or gonadal and thyroid hormone concentrations. Moreover, leptin participates in cell proliferation and immune cell activation. Leptin regulates appetite, controls energy stores, and impacts homeostasis, memory capabilities, and emotions [23]. It is known that leptin has a neuroprotective value. Leptin protects from dopaminergic neuron loss caused by 6-hydroxydopamine (6-OHDA). Furthermore, it decreases the activity of caspases 3 and 9, as well as other markers of apoptosis [24]. Changes in leptin concentrations could be considered a marker of various neurodegenerative diseases or mental disorders [22]. Correlations between serum leptin level and PD have been studied before, but all research results show that it is not statistically significant [6,25]. Nowadays, human recombinant leptin, along with various potent agonists and antagonists, are widely accessible. In 2018, the European Medicines Agency (EMA) and the Food and Drug Administration (FDA) approved human recombinant leptin for the treatment of lipodystrophy associated with leptin deficiency. This therapy has demonstrated a sustained effect on hypertriglyceridemia and glycemic control, and has been shown to have a favorable safety profile [26].

As an adipokine, resistin has a pro-inflammatory role in the human body; therefore, the disease progression might affect its levels in PD [27]. Resistin is a small secreted protein produced during adipocyte differentiation [28]. It belongs to a family of cysteine-rich proteins with a unique tissue distribution [29]. Its main circulating human sources include peripheral blood mononuclear cells (PBMCs), macrophages, and bone marrow. However, circulating resistin is also synthesized in the pituitary gland, hypothalamus, epithelial cells from the gastrointestinal tract, goblet cells, adrenal glands, skeletal muscle, pancreas, spleen, placenta, and synovial tissue [30]. It is found in two conformations: an oligomer of 660 kDa and a trimer with a molecular weight of 45 kDa [31]. Resistin regulates glucose and lipid metabolism, induces insulin resistance, modulates central nervous system (CNS) cells, and is associated with the synthesis and secretion of pro-inflammatory cytokines [28]. Increasing resistin levels cause a shift into a proinflammatory state [32]. The inflammatory process is a crucial pathophysiological feature of neurodegeneration [33]. Resistin promotes the secretion of tumor necrosis factor-alpha (TNF-α) and interleukin 1β (IL-1β), IL-6, IL-8, and IL-12, and it induces the generation of reactive oxygen species (ROS). It is also responsible for the release of monocyte chemotactic protein-1 (MCP-1), as well as the release of nuclear factor kappa-light-chain-enhancer of activated B cells (NF-κB) [30,34]. Resistin also plays a role in the functioning of neural stem cells. It inhibits astrocyte differentiation due to the mediation of toll-like receptor 4 (TLR-4) and CD36, which increases blood–brain barrier (BBB) permeability [35]. 

One of the best-known adipokines is also adiponectin. It reduces chronic inflammation in target organs by inhibiting macrophage differentiation, switching the macrophage function to an anti-inflammatory state, decreasing expression of TLR4, and modifying inflammation in a variety of cell types [36]. Adiponectin stimulates the release of IL-10, an anti-inflammatory cytokine. The anti-inflammatory effects of adiponectin in macrophages, endothelial cells, cardiomyocytes, and fibroblasts make it protective for the vasculature, heart, lung, and colon. Adiponectin performs this action, at least in part, by inhibiting TNF-α-induced expression of adhesion molecules, namely vascular cell adhesion molecule-1 (VCAM-1), endothelial–leukocyte adhesion molecule-1 (E-selectin), and intercellular adhesion molecule-1 (ICAM-1) [37]. Adiponectin is inversely associated with metabolic syndrome and obesity [38]. The adiponectin concentration depends on circadian rhythms, with serum levels higher during a day [39]. Peroxisome proliferator-activated receptor gamma (PPARγ), expressed mainly in adipose tissue, is the major positive regulator of adiponectin gene expression [4]. Insulin-like growth factor (IGF-1) and growth hormone positively regulate adiponectin gene expression and secretion in murine and human adipose tissue [40]. Pro-inflammatory mediators, such as TNF-α and IL-6, inhibit adiponectin secretion [41].

In the CNS, adiponectin is expressed in several different types of neurons and is expected to perform regulatory roles in animal behaviors. Thus, it may be a potential therapeutic target in neurodegenerative and neuropsychiatric disorders [42]. As a result of recent discoveries, adiponectin has been shown to regulate neural functions [43,44]. It regulates neural plasticity in the hippocampus at different levels, mediating neurogenesis [45]. Moreover, adiponectin mediates neurogenesis partly by activating PPAR-y [46]. Dysregulation of adiponectin signaling affects synaptic plasticity and accelerates neurodegeneration. It is thought that adiponectin can affect the synaptic morphology by other pathways based on its effects on insulin sensitivity [46]. Adiponectin can decrease neurotoxicity by suppressing the activation of NF-κB, an inflammatory factor contributing to neurodegeneration [47]. Adiponectin decreases the expression of pro-inflammatory cytokines such as TNF-α and increases the expression of anti-inflammatory molecules such as IL-10. Pro-inflammatory cytokines, including TNF-α and IL-6, inhibit adiponectin production [48]. These data also suggest an anti-inflammatory effect on adiponectin signaling. Chronic low-grade neuroinflammation seems to be correlated with neurodegenerative disorders such as PD [49].

Considering the above-mentioned data concerning the possible role of melatonin, leptin, resistin, and adiponectin in neurodegeneration processes, this study aimed to compare the concentration of these compounds in PD patients with and without dyskinesia symptoms and in healthy people. Given the lack of available data in the literature regarding melatonin and adipokine levels in PD patients at various stages of the disease (from mild to advanced), this study aimed to find the correlations between these parameters and the stage of the disease. It may be helpful in modifying and predicting therapy as it pertains to dyskinesia in patients suffering from PD. Enhanced comprehension of the neuroprotective function of melatonin and adipokines in PD could potentially lead to the development of disease-modifying therapies.

## 2. Material and Methods

In this study, 44 PD patients, qualified into two groups by their score in the Hoehn–Yahr scale [50], which is used to assess the severity of PD and the presence of dyskinesia, have been compared. The first group consisted of 20 patients without dyskinesia, and the second group contained 24 patients with dyskinesia. Additionally, the control group of 20 healthy volunteers has been examined. The groups were matched for sex, age, and body mass index (BMI). Patients differed in elapsed time from the diagnosis of PD. Table 1 presents the anthropometric and clinical data of the subjects enrolled in the study. The study was approved by the Bioethics Committee of the Nicolaus Copernicus University in Toruń functioning at Collegium Medicum in Bydgoszcz, Poland (consent no. KB 184/2015). All subjects gave written consent to participate in the study.

Both study groups received proper pharmacological treatment. The medication prescribed by a specialist physician for each patient was a combination of benserazide with levodopa. Benserazide is an inhibitor of extra-cerebral aromatic amino acid decarboxylase. Thanks to this component, levodopa achieves greater bioavailability in the target cells of the CNS. Table 2 shows the treatment regimen for PD in the study groups.

The blood samples were obtained from the “Neuromed” Medical Center in Bydgoszcz, Poland. Qualified medical personnel collected blood samples from the median cubital vein between 7:00 a.m. and 9:00 a.m. after an overnight fast. A 6 mL polypropylene tube containing a clot activator and gel separator (Greiner Bio-One GmbH, Kremsmünster, Austria) was used to collect each blood sample. The blood samples were centrifuged at 6000× *g* for 10 min at 4 °C to separate the blood serum. Then, the blood serum was aliquoted and placed in Eppendorf tubes (Eppendorf SE, Hamburg, Germany). The serum samples were preserved at −80 °C in the Department of Medical Biology and Biochemistry, Faculty of Medicine, Ludwik Rydygier Collegium Medicum in Bydgoszcz at Nicolaus Copernicus University in Toruń, Poland, for subsequent biochemical analysis. Serum concentrations of melatonin, resistin, leptin, and adiponectin were measured using commercially available corresponding enzyme-linked immunosorbent (ELISA) assays, including ELISA Kit for Melatonin (Cloud-Clone Corp., Wuhan, China), Human Resistin (BioVendor, Brno, Czech Republic), Human Leptin (BioVendor, Brno, Czech Republic), and Human Adiponectin (BioVendor, Brno, Czech Republic). The basis of the test is antigen binding by specific antibodies in the wells of the microplates of the kit. Melatonin concentrations were expressed in pg/mL, resistin and leptin in ng/mL, and adiponectin in µg/mL. Hematological parameters were examined with the automatic analyzer MYTHIC 22 AL (Orphée, Plan-les-Ouates, Switzerland).

Statistical analysis was performed using Statistica 13.3 (TIBCO Software Inc., Palo Alto, CA, USA) and Python 3.8.10 (Python Software Foundation, Wilmington, DE, USA) using pandas (v. 1.4.3) and scipy (v. 1.10.1) libraries. The results were presented as means, standard error of the mean (SEM), median, and interquartile range (IQR). Equivalence of the groups was checked with the chi-square test, normality with the Shapiro–Wilk test, and equality of variances with the Levene’s test. If the statistical analysis showed that the results met the assumptions of normal distribution and equality of variances, a one-way ANOVA with the Tukey HSD post hoc test was performed. If the criterion of normal distribution was met and the criterion of equal variance was not met, a one-way ANOVA with post hoc T2 Tamhane test was performed. When the results did not meet the criterion of normal distribution, the Kruskal–Wallis test with post hoc Mann–Whitney U analysis with the Bonferroni correction was performed. This study utilized the Pearson’s correlation coefficient to examine relationships between measured parameters. The level of significance was set at *p* < 0.05. 

## 3. Results

**Figure 1:**
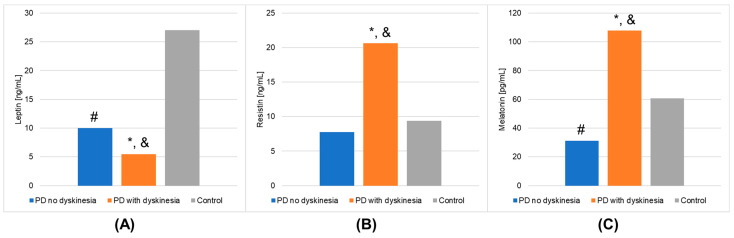
Outcomes of biochemical analyses (mean values) that demonstrated statistically significant differences in the Parkinson’s disease (PD) groups and healthy volunteers (control group). (**A**): leptin; (**B**): resistin; (**C**): melatonin. A value of *p* < 0.05 was considered statistically significant. If *p* < 0.05: * PD no dyskinesia vs. PD with dyskinesia; # PD no dyskinesia vs. control; & PD with dyskinesia vs. control.

**Figure 2:**
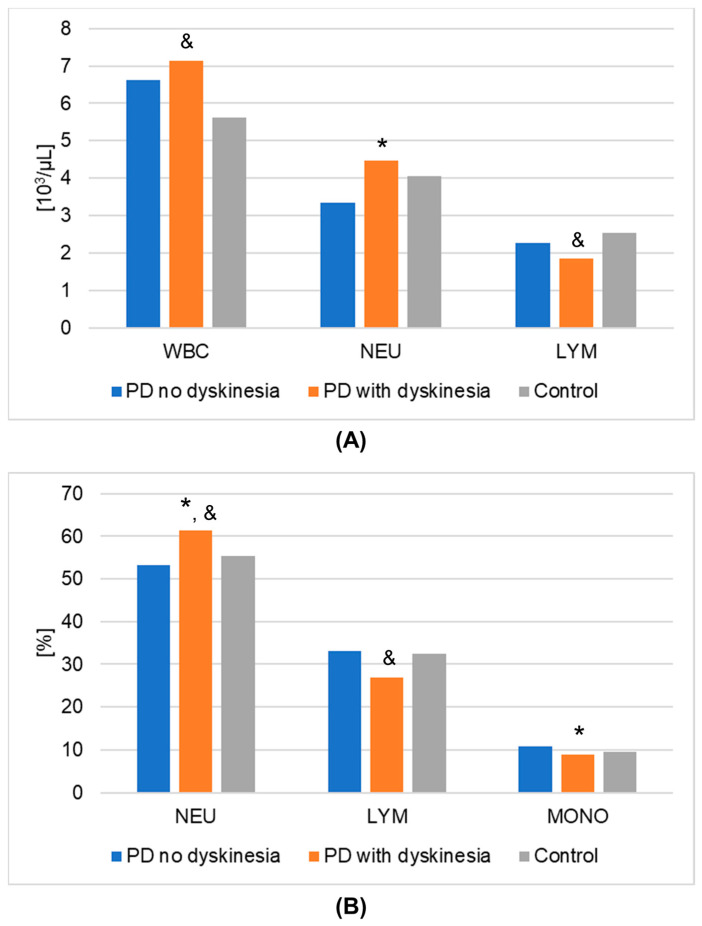
Results of hematological analyses (mean values) that demonstrated statistically significant differences in Parkinson’s disease (PD) groups and healthy volunteers (control group). (**A**): numeric values; (**B**): percentages. A value of *p* < 0.05 was considered statistically significant. If *p* < 0.05: * PD no dyskinesia vs. PD with dyskinesia; & PD with dyskinesia vs. control. Abbreviations used: LYM—lymphocytes; MONO—monocytes; NEU—neutrophils; WBC—white blood cells.

The results of laboratory determinations are presented in Table 3. There were statistically significant differences between the studied groups in the leptin, resistin, and melatonin concentrations. For resistin and melatonin, lower concentrations were observed in patients without dyskinesia than those with dyskinesia. Interestingly, leptin levels were significantly lower in patients with dyskinesia. At the same time, the concentrations of leptin and melatonin in the patients without dyskinesia were significantly lower than in the controls. It is worth mentioning that the differences between the studied groups in the determinations of white blood cells, mean platelet volume, % neutrophils, % lymphocytes, % monocytes, neutrophils, and lymphocytes also reached statistical significance. The results of the post hoc analysis are presented in Table 4. Figure 1 shows the results of biochemical analyses that showed statistically significant differences. Figure 2 displays the outcomes of hematological analyses, which revealed statistically significant differences.

**Figure 3:**
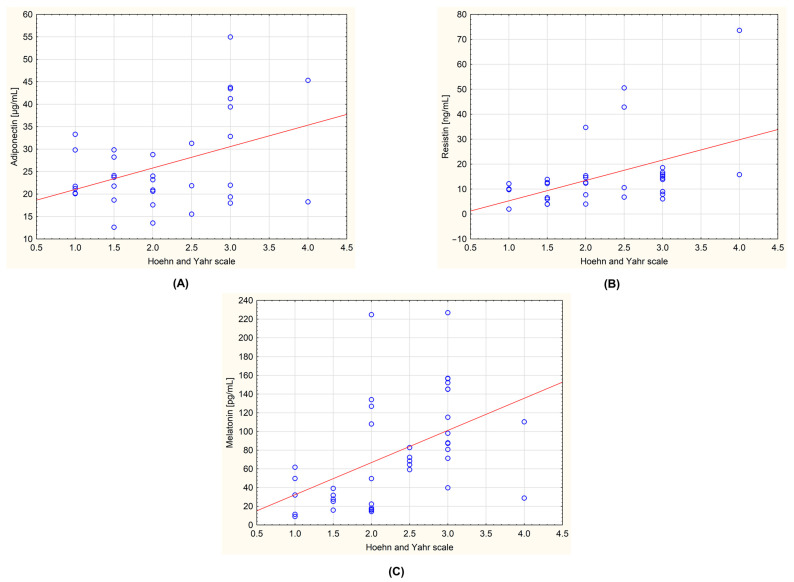
Correlations between the measured biochemical parameters and the severity of the Parkinson’s disease evaluated by Hoehn–Yahr scale in the group of all examined patients (*n* = 44). (**A**): adiponectin and Hoehn–Yahr scale (r = 0.408; *p* = 0.017); (**B**): resistin and Hoehn–Yahr scale (r = 0.477; *p* = 0.004); (**C**): melatonin and Hoehn–Yahr scale (r = 0.478; *p* = 0.002). The regression line is marked with a solid line. A value of *p* < 0.05 was considered statistically significant.

The obtained data were also tested for the presence of correlations between the level of the biochemical parameters and the stage of the disease determined by the Hoehn–Yahr scale. In the group of all patients with PD, a statistically significant positive correlation was observed for adiponectin and Hoehn–Yahr scale (r = 0.408; *p* = 0.017), resistin and Hoehn–Yahr scale (r = 0.477; *p* = 0.004), and melatonin and Hoehn–Yahr scale (r = 0.478; *p* = 0.002). These correlations are shown in Figure 3. An analysis was also carried out, taking into account the division of the patients into the subgroups with and without dyskinesia. No statistically significant correlations were observed.

## 4. Discussion

**Figure 4:**
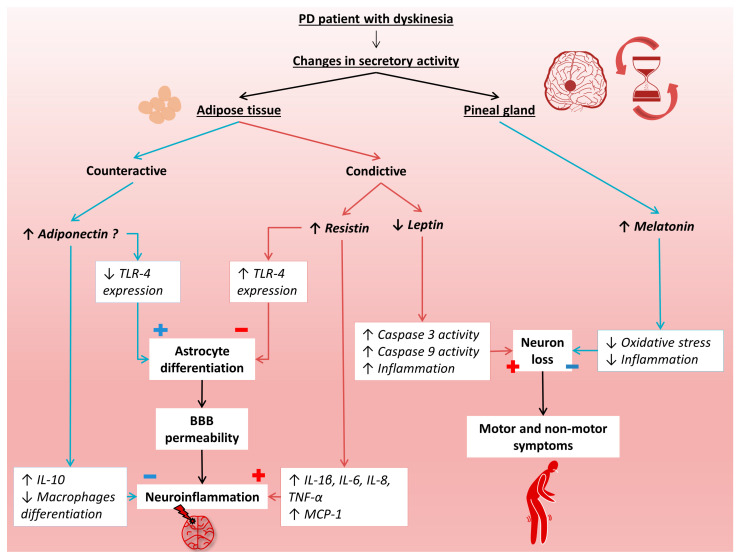
Putative mechanisms of melatonin, resistin, leptin, and adiponectin participation in the progression of Parkinson’s disease. BBB, blood–brain barrier; IL-1 β, interleukin 1β; IL-6, interleukin 6; IL-8, interleukin 8; IL-10, interleukin 10; MCP-1, monocyte chemoattractant protein 1; PD, Parkinson’s disease; TNF-α, tumor necrosis factor α; TLR-4, toll- like receptor 4.

As mentioned earlier, PD is a common neurodegenerative disease that has a detrimental impact on the patient’s quality of life. Despite standard treatment with levodopa, the disease progresses, leading to irreversible deterioration of the patient’s condition, mainly in the form of dyskinesias. In the development of PD, disturbed circadian rhythms can be observed, which may be associated with abnormal secretion of melatonin and adipokines produced in adipose tissue. However, there are limited data on melatonin and adipokines in the course of PD. Therefore, in the presented study, the level of melatonin, resistin, leptin, and adiponectin in the early and advanced stages of PD in relation to healthy people were examined. Figure 4 presents the putative mechanisms involved in the progression of PD by these hormonally active molecules. 

In the presented study, the serum levels of melatonin and leptin were statistically significantly lower in the PD patients without dyskinesia compared to the control group. Moreover, resistin concentrations were statistically significant increased, whereas leptin level was decreased in the dyskinesia PD patients versus the control group. Surprisingly, a higher melatonin concentration and a lower leptin concentration were found in the patients in the advanced stage of the disease as compared with the early stage.

Melatonin is a neurohormone synthesized by the pineal gland, but it is also produced, e.g., in the skin and intestine [14]. The release of melatonin is synchronized with the circadian rhythm. Its greatest concentration is observed during the night [51,52]. In the evening, serum melatonin levels rise, peaking at around 2–4 a.m., then decline again until the daily low levels are reached. Melatonin production is suppressed by light, which reduces the level of this compound during the day [53]. In the blood serum in the evening hours, melatonin reaches the value between 80 and 120 pg/mL, then drops to 10 to 20 pg/mL in the morning hours [54]. The melatonin level depends on age and is lower in old age [55]. Melatonin concentration in infancy is very low but gradually increases during childhood. After one year, the value may increase to 250 pg/mL [56]. Between the age of 65 and 75, the average melatonin level is estimated at around 49 pg/mL. After age 75, its mean value decreases to 28 pg/mL. In the elderly, during the day time, melatonin concentration drops and ranges from 10 to 20 pg/mL [57]. Melatonin presents a pleiotropic effect, having anti-inflammatory and antioxidant properties. It works as a direct free radical scavenger [58]. Moreover, it acts as an indirect antioxidant by stimulating the synthesis of antioxidant enzymes and inhibiting pro-oxidant enzymes [59]. Kakhaki et al. [8] evaluated the impact of melatonin supplementation on clinical and metabolic profiles in people with PD. Their study confirmed the favorable effects of 12 weeks of melatonin supplementation on patients with PD. The supplementation significantly reduced the Unified Parkinson’s Disease Rating Scale (UPDRS) part I score, Pittsburgh Sleep Quality Index (PSQI), Beck Depression Inventory, and Beck Anxiety Inventory (BAI) compared with the placebo treatment. They also found a significant reduction in serum high-sensitivity C-reactive protein (hs-CRP) levels and a significant increase in plasma total antioxidant capacity (TAC) and total glutathione (GSH) levels. Considering the nature of PD, which results in the death of dopaminergic cells and excessive oxidative stress, it can be argued that melatonin has mitigating properties against these phenomena. In the presented study, morning levels of melatonin in the blood serum were determined. The melatonin concentration of 60.6 ± 4.84 pg/mL was found in the healthy subjects, and a mean value of 31.2 ± 6.26 pg/mL was reached in the group of patients without dyskinesia; whereas, in the patients with dyskinesia, the mean score was set at 107.7 ± 10.72 pg/mL. The patients in the early stage of PD had about 2 times lower melatonin concentration than the healthy people, which confirms the influence of disturbed melatonin secretion in the development of this neuropathology. However, the patients with advanced-stage PD revealed about 3.5 times higher melatonin concentration than non-advanced-stage PD patients. Moreover, a positive correlation was observed between melatonin and the Hoehn–Yahr scale in the presented study. Thus, it could be concluded that the concentration of melatonin in the blood serum increases with the progression of the disease. Breen et al. [60] observed lower melatonin levels in PD patients than in healthy volunteers and correlated these decreased levels with the hypothalamic gray matter volume and severity of the disease. Considering the melatonin function in the human body, one might argue that its increased concentration counteracts adverse metabolic changes in PD patients, such as excessive apoptosis of neuronal cells. It is plausible that the elevated melatonin levels in advanced PD might be due to cellular apoptosis, which acts as a mechanism of prevention [51]. The increased melatonin concentration in patients with neurodegenerative diseases can also be explained by its antioxidant effect, which positively influences neurons by reducing the damage inflicted by ROS [61]. In the presented study, all subjects were given a standard PD treatment with levodopa, the precursor of dopamine. In our opinion, the treatment time with this drug may be connected with increased melatonin levels in PD patients with dyskinesia. In general, levodopa therapy begins as soon as PD is diagnosed; therefore, the time from diagnosis may be an approximate date for initiating this therapy. Thus, the median duration of levodopa treatment in the patients with dyskinesia may be about 8.2 years and about 1.3 years for the patients without dyskinesia. Similarly to the results of the presented study, Lin et al. [62] found a positive correlation between melatonin concentration and the disease progression based on the HY scale. This finding is not unexpected, as dopamine plays a crucial role in regulating the circadian rhythm, and circadian rhythms also affect dopamine metabolism [63]. The circadian system regulates the synthesis, release, and clearance of dopamine, which in turn affects sleep and wakefulness, as well as other physiological processes. Furthermore, the disruption of the circadian rhythm can lead to alterations in dopamine levels, which may contribute to the development or exacerbation of neurodegenerative disorders such as Parkinson’s disease. Therefore, understanding the interaction between dopamine and the circadian system is essential for developing effective treatments for sleep and other circadian rhythm disorders in Parkinson’s disease. However, no difference was found between the level of melatonin in elderly patients and those with PD in another research [64]. The phase angle of entrainment is a term used to describe the time difference between the onset of an external time cue, such as light exposure, and the onset of a biological rhythm, such as the secretion of melatonin. It reflects the ability of the biological clock to adjust to changes in the external environment and synchronize with the 24-h day-night cycle [63]. According to Bolitho et al. [65], medicated PD patients showed a prolonged phase angle of entrainment of the melatonin rhythm compared to the PD unmedicated group and controls. These findings suggest that there may be an uncoupling of circadian and sleep regulation, possibly related to dopaminergic therapy. The limitation of the presented result may include the time of blood sample collection as in the presented study it was collected in the morning. That problem has not been fully solved by any of the existing studies within this field, so further research is necessary. At the moment, the results of different studies concerning melatonin levels in the course of PD are inconclusive. More research is required to provide more detailed insight into this neurodegenerative disease and the effect of melatonin on metabolic and oxidative changes in PD. However, some studies suggest that melatonin may improve sleep quality and reduce daytime sleepiness in Parkinson’s disease patients, and its effects on motor symptoms and the disease progression are still unclear. Nonetheless, the presented findings open up the possibility of early identification of sleep problems, which could potentially reduce the motor impairment in individuals with PD. Additionally, there is a lack of standardized dosing and administration protocols for melatonin in Parkinson’s disease, with different studies using different doses and routes of administration. Moreover, melatonin treatment in Parkinson’s disease may have potential side effects and drug interactions. For example, melatonin can increase the sedative effects of some medications, such as benzodiazepines and opioids, and may also affect blood pressure and blood sugar levels. Further studies are needed to determine the safety and potential risks of melatonin treatment in Parkinson’s disease.

In the presented study, a correlation between plasma leptin levels in the PD patients and disease progression in the form of dyskinesia was determined. Leptin is associated primarily with homeostatic regulation. It is transported from the periphery to the brain through the BBB, acting on various CNS structures. Its effects on the hypothalamus, activities such as thermogenesis, and synaptic transmission have been described [24]. Moreover, it has been shown to have an anti-apoptotic effect. Its quantitative deficit and the resulting decreased neuroprotection may be a component of the clinical picture of PD progression and the appearance of dyskinesia. Leptin is also known to reduce the action of pro-inflammatory cytokines, one of the culprits of progressive neurodegeneration. It also has a protective effect in the case of factors strictly neurotoxic for the CNS, like 6-OHDA. Leptin regulates the homeostatic functioning of the nigrostratial pathway [24]. Moreover, leptin reduces the activity of pro-apoptotic factors, including caspase 3 and caspase 9. In PD, reduced expression of leptin and insulin receptors has been found, which might be involved in the pathomechanism of the disease, similarly to Alzheimer’s disease and other neurodegenerative diseases [24]. The leptin receptor and leptin itself also play an essential role in the proper functioning of the limbic system in creating emotions. These, in turn, are disturbed during PD [25]. On the other hand, chronically elevated levels of leptin can cause N-methyl-D-aspartate (NMDA) receptor-mediated depression [66]. It is also worth considering other aspects related to links between leptin and PD. Various correlations between this adipokine and the incidence and course of the disease have been examined. In previous studies, the average leptin level in younger patients was lower than in the elderly [67]. It is worth mentioning that high, statistically significant differences between blood leptin levels in both sexes were reported in the study by Fiszer et al. [68]. Women likely have higher adipokine levels due to the greater average proportion of subcutaneous fat deposits than men. Moreover, the mentioned difference may be caused by the specific influence of female steroid hormones inducing increased leptin expression [67]. High BMI is associated with the limited availability of leptin, which may particularly exacerbate the disorders of dopamine-related neurotransmission and its deficiency. Obese people have a reduced number of dopamine receptors in the striatum. Disturbances in the hormonal transmission of energy information due to leptin deficiency may be one of the culprits behind unintentional weight loss in PD patients [68]. Tremors and other motor symptoms in patients may likely explain weight loss and lipid imbalance. NMS, such as dysphagia, anorexia, and depression-induced dysphagia, should also be considered, as they contribute mainly to weight loss [67]. Interestingly, PD patients with unintentional weight loss report higher satiety levels after meals than healthy controls. An overall decreased appetite has been also observed [69]. Patients with PD usually begin to lose weight a few years before clinical diagnosis [70]. The use of L-dopa and drugs from the dopamine agonist group indirectly results in decreased serum leptin concentrations and is associated with weight disorders [67]. Lorefält et al. [25] found no correlation between L-dopa use and serum leptin levels. Researchers point out, however, that the metabolic changes caused by taking the drug may lead to decreased subcutaneous fat deposits, thus reducing leptin concentration. Adams et al. [71] showed that levodopa attenuates isoproterenol-induced lipid mobilization by a specific metabolism modulation without changing the blood flow in the adipose tissue. Dopaminergic therapy is associated with excessive lipolysis resulting from hyperinsulinemia, ultimately causing weight loss [72]. L-dopa reduces blood flow to skeletal muscles. There is also a reduction in glucose uptake by skeletal muscle. Such treatment directs glucose from the synthesis of muscle glycogen to aerobic glycolysis. A reduction in the lactate-to-pyruvate ratio with L-dopa leads to an increase in the size of aerobic glycolysis [71]. Given the accumulating evidence of the various roles that leptin plays in the etiology and progression of Parkinson’s disease, which includes its ability to modulate neuroinflammation, it is imperative to conduct further studies that validate leptin and other adipokines as potential therapy target. As the statistically significant decrease of leptin was observed even at early stage of the disease, it would be interesting to explore whether leptin may be a potential biomarker for Parkinson’s disease, potentially aiding in earlier diagnosis or disease monitoring.

Resistin, as an adipokine, is synthesized primarily by adipocytes. Additionally, its high secretion is observed in monocytes and macrophages [73]. Current research is ambiguous about whether adipocytes or macrophages are the primary sources of resistin in people [74]. Due to its pro-inflammatory nature, manifested by the activation of TNF-α, interleukins, and promotion of oxidative stress, resistin is considered to be involved in pathologies such as obesity, insulin resistance, neoplasia, and atherosclerosis [35,75]. Physiological serum levels of resistin range from 7 to 22 ng/mL [28]. In PD patients, serum resistin concentration may vary due to metabolic and pro-inflammatory changes. In the study by Rocha et al. [27], no changes in resistin levels were observed in PD patients compared to healthy subjects. In their study, no correlation between PD and non-PD patients was found. This is in line with the results of the presented study which determined no differences in resistin levels between the patients with the early stage of PD and the healthy subjects. However, in the presented study, a correlation between resistin concentration and the severity of PD was observed. In the study group without dyskinesia, the resistin level was lower compared to the study group with dyskinesia. The patients with advanced-stage PD had approximately 2.7 times higher concentrations of resistin than the non-advanced-stage PD patients. Moreover, resistin concentrations correlated positively with the HY scale. These findings seem to confirm the negative role of resistin as a pro-inflammatory adipokine in the processes associated with the advanced stage of PD. It could be concluded that resistin participates in the progression of neurodegeneration during PD and may be a factor related to the development of dyskinesia. Thus, resistin might be a target in the prevention treatment against the development of dyskinesia in PD patients. Undoubtedly, further research is required to explain the possible mechanisms of resistin role in the progression of PD.

In the presented study, adiponectin levels in the PD patients were found to be unaltered compared to the controls. Literature data suggest that decreased secretion of this anti-inflammatory adipokine might participate in the development of chronic neuroinflammation associated with the pathogenesis of neurodegenerative disorders such as PD [47]. Adiponectin induces the production of critical anti-inflammatory cytokines, such as IL-10 and the IL-1 receptor antagonist (IL-1RA), by human monocytes, macrophages, and dendritic cells and suppresses the production of interferon-γ (IFN-γ) [76]. Neuroinflammatory mechanisms contribute to the pathology of PD, comprising microglia activation, astrocytosis, and lymphocyte infiltration as observed in post-mortem obtained brain tissue [77]. Adiponectin receptors are expressed in the hypothalamus, brainstem, and basal ganglia [78]. Sekiyama et al. [79] observed that adiponectin is localized in Lewy bodies derived from α-synucleinopathies, such as PD and dementia with Lewy bodies. They also noted that in neuronal cells expressing α-synuclein, aggregation of α-synuclein was suppressed by treatment with recombinant adiponectin in an AdipoRI-AMP kinase pathway-dependent manner. PD is caused by decreased dopaminergic neurons and α-synuclein accumulation in the substantia nigra. Thus, the adiponectin may suppress neurodegeneration through a modification of the metabolic pathway and could possess a therapeutic potential against α-synucleinopathies [79]. Therefore, it seems as though adiponectin would protect against neurodegenerative disease and its progression. In the presented study, no differences in adiponectin level were found in the PD patients with dyskinesia compared to the patients in the early stage of the disease. Adiponectin is an adipokine inversely correlated with body mass. As a result, adiponectin is increased during periods of weight loss, which is also a symptom of PD [49]. Dyskinesia has been also found to be inversely correlated with body mass and positively correlated with weight loss [47]. Nevertheless, it should be noted that the studied groups of patients had similar values of BMI. The mechanisms associated with dyskinesia seem to be correlated with pulsatile dopamine agonism and the degeneration of nigral cells in the basal ganglia [80]. The extent of these pathologies is more likely severe in patients with longer disease duration. It should be emphasized that adiponectin concentrations in the cerebrospinal fluid (CSF) were not measured in the presented study, and the levels measured in blood serum cannot be directly translated into concentrations of this adipokine in the CNS because some adiponectin multimers, such as HMW adiponectin, cannot cross the BBB [81]. Cassani et al. [82] determined the adiponectin concentration in Parkinson’s disease patients treated with levodopa and a dopamine agonist for neurological symptoms. They found that in PD patients, adiponectin serum levels were similar to those in normal-weight, healthy, young subjects and significantly higher than those in an aged-matched group of morbidly obese subjects.

The main limitation of the presented study is the relatively small number of the participants, i.e., 44 subjects suffering from PD, including 20 patients without dyskinesia and 24 patients with dyskinesia. Therefore, the results might not be strictly representative of the larger population. The limited number of participants in the study is a consequence of the restrictive inclusion and exclusion criteria that were used to make the groups as homogenous as possible. Obviously, with small sample sizes, it could be challenging to ensure that the participants are truly representative of the population being studied, which can lead to biased results. Additionally, with fewer participants, the statistical power of the study may be reduced, making it difficult to detect significant differences. However, in the presented study, an advanced statistical analysis was performed, and statistically significant differences were confirmed. 

Although the secretion of numerous adipokines has been investigated in relation to Parkinson’s disease, there is still a lack of studies exploring the potential interaction between these factors and how they may affect the progression of the disease. Specifically, more research is needed to determine if the observed changes in the secretion of these substances are simply a consequence of Parkinson’s disease or if they play a more active role in the pathophysiology of PD. Undoubtedly, further research on a larger group of patients, enabling their long-term observation during the disease progression, is crucial to obtaining reliable results that could inspire clinical trials on modifying the secretion of melatonin and the tested adipokines for the purpose of PD therapy,

## 5. Conclusions

The obtained results indicate that melatonin is a hormone whose reduced secretion might be involved in the development of Parkinson’s disease. Thus, symptoms characteristic of early-stage PD, including sleep disorders, might be related to the melatonin deficiency. Melatonin supplementation could be recommended in the prevention of PD and in reducing the troublesome symptoms of early PD. Surprisingly, high melatonin levels were found to positively correlate with the advanced PD evaluated by the Hoehn–Yahr scale. This could be a consequence of prolonged use of levodopa in the treatment of this neurodegenerative disease, but further research is needed to explain these relationships. Moreover, impaired adipokine secretion was observed, including leptin and resistin. Low leptin levels appear to be involved in both the development of PD and the disease progression. Interestingly, resistin concentrations were elevated only in the PD dyskinesia patients, which indicates the potential role of this adipokine in the progression of PD leading to dyskinesia. The changes in the concentration of adiponectin have not reached statistical significance, but its level positively correlated with the severity of the disease as assessed by the Hoehn–Yahr scale. Summing up, the presented results indicate the possible involvement of melatonin and the studied adipokines in both the development of PD and the progression of the disease. We hope that our research may inspire the scientific community dealing with this socially important disease to undertake further research on both the mechanisms of action of melatonin, adiponectin, resistin, and leptin in the course of PD and the possibility of developing new therapies in the prevention and treatment of PD related to these substances.

## Figures and Tables

**Table 1:** Anthropometric and clinical characteristics of the patients with Parkinson’s disease and healthy volunteers (control group). *p* < 0.05 was considered as statistically significant (marked in **bold**).

Parameter		Parkinson’s Disease		Control	*p* Value	Power of a Test
		No Dyskinesia	With Dyskinesia			
***n* (Female/Male)**		20 (9/11)	24 (10/14)	20 (8/12)	-	-
**Age [years]**	Mean	68.250	68.417	66.950	0.6220	0.1069
	SEM	1.326	1.302	0.540		
	Median	69.000	68.500	67.500		
	IQR	7.250	6.500	4.000		
**Body mass [kg]**	Mean	81.250	74.021	78.500	0.7065	1.0000
	SEM	4.852	3.147	2.162		
	Median	78.500	74.000	77.500		
	IQR	21.500	14.500	15.250		
**Height [cm]**	Mean	170.011	166.958	169.700	0.2443	0.2374
	SEM	1.761	1.372	1.093		
	Median	169.000	167.500	169.000		
	IQR	10.000	6.500	6.250		
**BMI [kg/m^2^]**	Mean	27.829	26.419	27.218	0.4982	0.9604
	SEM	1.214	1.006	0.608		
	Median	26.037	25.510	27.184		
	IQR	4.183	4.412	3.899		
**Hoehn–Yahr scale**	Mean	1.500	2.813	0.000	**<0.0001**	0.9999
	SEM	0.089	0.108	0.000		
	Median	1.500	3.000	0.000		
	IQR	1.000	0.500	0.000		
**Years since diagnosis [years]**	Mean	1.300	8.167	0.000	**<0.0001**	1.0000
	SEM	0.105	0.354	0.000		
	Median	1.000	8.000	0.000		
	IQR	1.000	3.250	0.000		

Abbreviations used: BMI: body mass index; IQR: interquartile range; SEM: standard error of mean.

**Table 2:** Pharmacological treatment in the examined groups of Parkinson’s disease (PD) patients.

Drugs, Dose	PD No Dyskinesia [*n* = 20]	PD with Dyskinesia [*n* = 24]
no treatment	0	0
madopar HBS	14	12
madopar 250 + HBS	0	7
madopar HBS + madopar 125	0	3
madopar HBS + madopar 125 + madopar 625	1	1
madopar 625 + HBS	4	0
madopar 125 + madopar 125	1	0
madopar x4	0	1

Active ingredients in medicines: madopar HBS: 100 mg levodopa + 25 mg benserazide; madopar 250 + HBS: 200 mg levodopa + 50 mg benserazide; madopar 125: 100 mg levodopa + 25 mg benserazide; madopar 125: 100 mg levodopa + 25 mg benserazide; madopar 62.5: 50 mg levodopa + 12.5 mg benserazide. The acronym HBS stands for a hydrodynamically balanced system.

**Table 3:** Biochemical and blood morphological parameters of the patients with Parkinson’s disease and healthy volunteers (control group). *p* < 0.05 was considered as statistically significant (marked in **bold**).

Parameter		Parkinson’s Disease		Control	*p* Value	Power of a Test
		No Dyskinesia	With Dyskinesia			
**Adiponectin [µg/mL]**	Mean	22.833	29.778	29.082	0.0626	0.9998
	SEM	1.486	2.824	1.981		
	Median	21.753	23.595	30.513		
	IQR	8.638	20.103	7.331		
**Leptin [ng/mL]**	Mean	9.983	5.431	27.030	**<0.0001**	1.0000
	SEM	1.414	0.933	2.860		
	Median	8.725	4.070	26.664		
	IQR	7.995	4.076	19.910		
**Resistin [ng/mL]**	Mean	7.768	20.645	9.392	**0.0013**	0.9082
	SEM	1.145	4.025	0.993		
	Median	7.140	14.640	9.143		
	IQR	8.188	6.159	4.724		
**Melatonin [pg/mL]**	Mean	31.205	107.742	60.580	**<0.0001**	0.9997
	SEM	6.256	10.719	4.844		
	Median	23.841	93.025	60.292		
	IQR	17.858	74.497	31.985		
**WBC [10^3^/µL]**	Mean	6.635	7.150	5.625	**0.0245**	0.5693
	SEM	0.511	0.388	0.211		
	Median	6.570	6.725	5.350		
	IQR	2.195	2.025	1.475		
**RBC [10^6^/μL]**	Mean	4.641	4.622	4.382	0.1117	0.3545
	SEM	0.097	0.093	0.090		
	Median	4.715	4.620	4.370		
	IQR	0.480	0.762	0.283		
**Hb [g/dL]**	Mean	14.225	14.154	13.865	0.6297	0.1053
	SEM	0.232	0.295	0.273		
	Median	14.500	14.000	14.100		
	IQR	1.425	2.025	1.600		
**HCT [%]**	Mean	42.090	40.421	40.560	0.1383	0.9929
	SEM	0.686	1.747	0.396		
	Median	42.850	41.150	40.200		
	IQR	4.475	4.800	1.375		
**MCV [fL]**	Mean	90.915	89.504	90.135	0.9075	0.9579
	SEM	0.817	2.013	1.000		
	Median	91.000	91.900	91.300		
	IQR	3.750	5.125	4.400		
**MCH [pg]**	Mean	30.700	30.588	31.050	0.3596	0.2495
	SEM	0.273	0.291	0.341		
	Median	30.700	30.700	31.200		
	IQR	1.150	1.675	1.825		
**MCHC [g/dL]**	Mean	33.765	33.471	33.767	0.1925	0.1517
	SEM	0.162	0.174	0.316		
	Median	33.700	33.400	34.055		
	IQR	0.850	0.625	1.666		
**RDW [%]**	Mean	13.710	14.079	14.115	0.2986	0.2080
	SEM	0.172	0.184	0.235		
	Median	13.600	13.900	14.150		
	IQR	0.875	1.150	0.850		
**PLT [10^3^/μL]**	Mean	234.450	217.667	254.250	0.0654	0.4336
	SEM	13.984	9.871	8.587		
	Median	216.500	211.000	246.000		
	IQR	83.500	81.750	59.750		
**MPV [fL]**	Mean	10.705	10.925	9.315	**<0.0001**	0.9889
	SEM	0.201	0.221	0.212		
	Median	10.900	10.850	9.050		
	IQR	1.250	1.375	1.150		
**% NEU [%]**	Mean	53.320	61.458	55.293	**0.0059**	0.7305
	SEM	2.446	1.669	1.242		
	Median	55.700	62.350	55.410		
	IQR	18.425	10.800	9.389		
**% LYM [%]**	Mean	33.075	26.925	32.541	**0.0168**	0.6166
	SEM	2.157	1.555	1.186		
	Median	33.350	24.750	31.589		
	IQR	18.800	11.450	6.511		
**% MONO [%]**	Mean	10.730	8.888	9.468	**0.0447**	0.9950
	SEM	0.647	0.615	0.431		
	Median	9.900	8.200	9.352		
	IQR	2.850	3.225	2.967		
**% EOS [%]**	Mean	2.740	2.196	2.362	0.6014	0.3214
	SEM	0.355	0.260	0.275		
	Median	2.400	2.050	2.276		
	IQR	2.450	1.100	1.444		
**% BASO [%]**	Mean	0.495	0.425	0.489	0.5830	0.0548
	SEM	0.080	0.069	0.057		
	Median	0.300	0.350	0.541		
	IQR	0.450	0.325	0.426		
**NEU [10^3^/μL]**	Mean	3.348	4.477	4.058	**0.0173**	0.8609
	SEM	0.257	0.271	0.288		
	Median	3.200	4.485	4.271		
	IQR	1.945	1.470	2.481		
**LYM [10^3^/μL]**	Mean	2.261	1.846	2.531	**0.0064**	0.7222
	SEM	0.166	0.137	0.148		
	Median	2.105	1.770	2.668		
	IQR	1.253	0.865	0.981		
**MONO [10^3^/μL]**	Mean	0.698	0.691	0.715	0.2321	0.0505
	SEM	0.043	0.080	0.051		
	Median	0.685	0.565	0.701		
	IQR	0.230	0.248	0.315		
**EOS [10^3^/μL]**	Mean	0.187	0.256	0.208	0.3068	0.0540
	SEM	0.025	0.099	0.022		
	Median	0.210	0.140	0.214		
	IQR	0.168	0.068	0.127		
**BASO [10^3^/μL]**	Mean	0.029	0.030	0.026	0.8760	0.0500
	SEM	0.003	0.003	0.001		
	Median	0.030	0.030	0.024		
	IQR	0.015	0.020	0.012		

Abbreviations used: % BASO: percent basophils; % EOS: percent eosinophils; % LYM: percent lymphocytes; % MONO: percent monocytes; % NEU: percent neutrophils; BASO: basophils; EOS: eosinophils; Hb: hemoglobin; HCT: hematocrit; IQR: interquartile range; LYM: lymphocytes; MCH: mean corpuscular hemoglobin; MCHC: mean corpuscular hemoglobin concentration; MCV: mean corpuscular volume; MONO: monocytes; MPV: mean platelet volume; NEU: neutrophils; PLT: thrombocytes; RBC: red blood cells; RDW: red blood cell distribution width; SEM: standard error of mean; WBC: white blood cells.

**Table 4:** Post hoc analysis results. *p* < 0.05 was considered as statistically significant (marked in **bold**).

Parameter	*p* Value		
	No Dyskinesia vs. with Dyskinesia	No Dyskinesia vs. Control	With Dyskinesia vs. Control
**Hoehn–Yahr scale**	**<0.0001**	**<0.0001**	**<0.0001**
**Years since diagnosis**	**<0.0001**	**<0.0001**	**<0.0001**
**Leptin**	**0.0360**	**<0.0001**	**<0.0001**
**Resistin**	**0.0030**	0.8922	**0.0064**
**Melatonin**	**<0.0001**	**0.0025**	**0.0010**
**WBC**	0.6085	0.1895	**0.0192**
**MPV**	1.0000	**0.0002**	**<0.0001**
**% NEU**	**0.0280**	0.8577	**0.0151**
**% LYM**	0.0777	0.9951	**0.0192**
**% MONO**	**0.0455**	0.6250	0.6476
**NEU**	**0.0120**	0.2033	1.0000
**LYM**	0.1262	0.4398	**0.0050**

Abbreviations used: % LYM: percent lymphocytes; % MONO: percent monocytes; % NEU: percent neutrophils; LYM: lymphocytes; MPV: mean platelet volume; NEU: neutrophils; WBC: white blood cells.

## Data Availability

Data are available on request due to privacy/ethical restrictions.

## References

[1] Armstrong M.J., Okun M.S. (2020). Time for a New Image of Parkinson Disease. JAMA Neurol..

[2] Armstrong M.J., Okun M.S. (2020). Diagnosis and Treatment of Parkinson Disease. JAMA.

[3] Tysnes O.-B., Storstein A. (2017). Epidemiology of Parkinson’s Disease. J. Neural Transm..

[4] Gustafson B., Jack M.M., Cushman S.W., Smith U. (2003). Adiponectin Gene Activation by Thiazolidinediones Requires PPARγ2, but Not C/EBPα—Evidence for Differential Regulation of the AP2 and Adiponectin Genes. Biochem. Biophys. Res. Commun..

[5] Stefanis L. (2012). Alpha-Synuclein in Parkinson’s Disease. Cold Spring Harb. Perspect. Med..

[6] Salari M., Barzegar M., Etemadifar M., Mirmosayyeb O. (2018). Serum Leptin Levels in Iranian Patients with Parkinson’s Disease. Iran. J. Neurol..

[7] Kalia L.V., Lang A.E. (2015). Parkinson’s Disease. Lancet.

[8] Daneshvar Kakhaki R., Ostadmohammadi V., Kouchaki E., Aghadavod E., Bahmani F., Tamtaji O.R., Reiter R.J., Mansournia M.A., Asemi Z. (2020). Melatonin Supplementation and the Effects on Clinical and Metabolic Status in Parkinson’s Disease: A Randomized, Double-Blind, Placebo-Controlled Trial. Clin. Neurol. Neurosurg..

[9] Ahn J.H., Kim M., Park S., Jang W., Park J., Oh E., Cho J.W., Kim J.S., Youn J. (2020). Prolonged-Release Melatonin in Parkinson’s Disease Patients with a Poor Sleep Quality: A Randomized Trial. Parkinsonism Relat. Disord..

[10] Hadi F., Agah E., Tavanbakhsh S., Mirsepassi Z., Mousavi S.V., Talachi N., Tafakhori A., Aghamollaii V. (2022). Safety and Efficacy of Melatonin, Clonazepam, and Trazodone in Patients with Parkinson’s Disease and Sleep Disorders: A Randomized, Double-Blind Trial. Neurol. Sci..

[11] Delgado-Lara D.L., González-Enríquez G.V., Torres-Mendoza B.M., González-Usigli H., Cárdenas-Bedoya J., Macías-Islas M.A., de la Rosa A.C., Jiménez-Delgado A., Pacheco-Moisés F., Cruz-Serrano J.A. (2020). Effect of Melatonin Administration on the PER1 and BMAL1 Clock Genes in Patients with Parkinson’s Disease. Biomed. Pharmacother..

[12] Jiménez-Delgado A., Ortiz G.G., Delgado-Lara D.L., González-Usigli H.A., González-Ortiz L.J., Cid-Hernández M., Cruz-Serrano J.A., Pacheco-Moisés F.P. (2021). Effect of Melatonin Administration on Mitochondrial Activity and Oxidative Stress Markers in Patients with Parkinson’s Disease. Oxid. Med. Cell. Longev..

[13] Wongprayoon P., Govitrapong P. (2017). Melatonin as a Mitochondrial Protector in Neurodegenerative Diseases. Cell. Mol. Life Sci..

[14] Alghamdi B.S. (2018). The Neuroprotective Role of Melatonin in Neurological Disorders. J. Neurosci. Res..

[15] Liu J., Clough S.J., Hutchinson A.J., Adamah-Biassi E.B., Popovska-Gorevski M., Dubocovich M.L. (2016). MT 1 and MT 2 Melatonin Receptors: A Therapeutic Perspective. Annu. Rev. Pharmacol. Toxicol..

[16] Videnovic A., Willis G.L. (2016). Circadian System—A Novel Diagnostic and Therapeutic Target in Parkinson’s Disease?. Mov. Disord..

[17] Tamtaji O.R., Reiter R.J., Alipoor R., Dadgostar E., Kouchaki E., Asemi Z. (2020). Melatonin and Parkinson Disease: Current Status and Future Perspectives for Molecular Mechanisms. Cell. Mol. Neurobiol..

[18] Ma H., Yan J., Sun W., Jiang M., Zhang Y. (2022). Melatonin Treatment for Sleep Disorders in Parkinson’s Disease: A Meta-Analysis and Systematic Review. Front. Aging Neurosci..

[19] Hu X., Li J., Wang X., Liu H., Wang T., Lin Z., Xiong N. (2023). Neuroprotective Effect of Melatonin on Sleep Disorders Associated with Parkinson’s Disease. Antioxidants.

[20] Zhang Y., Proenca R., Maffei M., Barone M., Leopold L., Friedman J.M. (1994). Positional Cloning of the Mouse Obese Gene and Its Human Homologue. Nature.

[21] Palhinha L., Liechocki S., Hottz E.D., da Pereira J.A.S., de Almeida C.J., Moraes-Vieira P.M.M., Bozza P.T., Maya-Monteiro C.M. (2019). Leptin Induces Proadipogenic and Proinflammatory Signaling in Adipocytes. Front. Endocrinol..

[22] Zou X., Zhong L., Zhu C., Zhao H., Zhao F., Cui R., Gao S., Li B. (2019). Role of Leptin in Mood Disorder and Neurodegenerative Disease. Front. Neurosci..

[23] Zhang Y., Chua S. (2017). Leptin Function and Regulation. Compr. Physiol..

[24] Signore A.P., Zhang F., Weng Z., Gao Y., Chen J. (2008). Leptin Neuroprotection in the CNS: Mechanisms and Therapeutic Potentials. J. Neurochem..

[25] Lorefält B., Toss G., Granérus A.-K. (2009). Weight Loss, Body Fat Mass, and Leptin in Parkinson’s Disease. Mov. Disord..

[26] Regensburger M., Rasul Chaudhry S., Yasin H., Zhao Y., Stadlbauer A., Buchfelder M., Kinfe T. (2023). Emerging Roles of Leptin in Parkinson’s Disease: Chronic Inflammation, Neuroprotection and More?. Brain. Behav. Immun..

[27] Rocha N.P., Scalzo P.L., Barbosa I.G., de Sousa M.S., Morato I.B., Vieira É.L.M., Christo P.P., Reis H.J., Teixeira A.L. (2014). Circulating Levels of Adipokines in Parkinson’s Disease. J. Neurol. Sci..

[28] Acquarone E., Monacelli F., Borghi R., Nencioni A., Odetti P. (2019). Resistin: A Reappraisal. Mech. Ageing Dev..

[29] Lu D.-Y., Chen J.-H., Tan T.-W., Huang C.-Y., Yeh W.-L., Hsu H.-C. (2013). Resistin Protects against 6-Hydroxydopamine-Induced Cell Death in Dopaminergic-like MES23.5 Cells. J. Cell. Physiol..

[30] Filková M., Haluzík M., Gay S., Šenolt L. (2009). The Role of Resistin as a Regulator of Inflammation: Implications for Various Human Pathologies. Clin. Immunol..

[31] Codoñer-Franch P., Alonso-Iglesias E. (2015). Resistin: Insulin Resistance to Malignancy. Clin. Chim. Acta.

[32] Jamaluddin M.S., Yan S., Lü J., Liang Z., Yao Q., Chen C. (2013). Resistin Increases Monolayer Permeability of Human Coronary Artery Endothelial Cells. PLoS ONE.

[33] Sardi F., Fassina L., Venturini L., Inguscio M., Guerriero F., Rolfo E., Ricevuti G. (2011). Alzheimer’s Disease, Autoimmunity and Inflammation. The Good, the Bad and the Ugly. Autoimmun. Rev..

[34] Silswal N., Singh A.K., Aruna B., Mukhopadhyay S., Ghosh S., Ehtesham N.Z. (2005). Human Resistin Stimulates the Pro-Inflammatory Cytokines TNF-α and IL-12 in Macrophages by NF-ΚB-Dependent Pathway. Biochem. Biophys. Res. Commun..

[35] Xiaoying L., Li T., Yu S., Jiusheng J., Jilin Z., Jiayi W., Dongxin L., Wengang F., Xinyue Z., Hao Y. (2019). Resistin-Inhibited Neural Stem Cell-Derived Astrocyte Differentiation Contributes to Permeability Destruction of the Blood–Brain Barrier. Neurochem. Res..

[36] Bruun J.M., Lihn A.S., Verdich C., Pedersen S.B., Toubro S., Astrup A., Richelsen B. (2003). Regulation of Adiponectin by Adipose Tissue-Derived Cytokines: In Vivo and In Vitro Investigations in Humans. Am. J. Physiol. Metab..

[37] Cnop M., Havel P.J., Utzschneider K.M., Carr D.B., Sinha M.K., Boyko E.J., Retzlaff B.M., Knopp R.H., Brunzell J.D., Kahn S.E. (2003). Relationship of Adiponectin to Body Fat Distribution, Insulin Sensitivity and Plasma Lipoproteins: Evidence for Independent Roles of Age and Sex. Diabetologia.

[38] Kadowaki T. (2006). Adiponectin and Adiponectin Receptors in Insulin Resistance, Diabetes, and the Metabolic Syndrome. J. Clin. Investig..

[39] Gavrila A., Chan J.L., Yiannakouris N., Kontogianni M., Miller L.C., Orlova C., Mantzoros C.S. (2003). Serum Adiponectin Levels Are Inversely Associated with Overall and Central Fat Distribution but Are Not Directly Regulated by Acute Fasting or Leptin Administration in Humans: Cross-Sectional and Interventional Studies. J. Clin. Endocrinol. Metab..

[40] Halleux C.M., Takahashi M., Delporte M.L., Detry R., Funahashi T., Matsuzawa Y., Brichard S.M. (2001). Secretion of Adiponectin and Regulation of ApM1 Gene Expression in Human Visceral Adipose Tissue. Biochem. Biophys. Res. Commun..

[41] Keller P., Møller K., Krabbe K.S., Pedersen B.K. (2003). Circulating Adiponectin Levels during Human Endotoxaemia. Clin. Exp. Immunol..

[42] Lihn A.S., Pedersen S.B., Richelsen B. (2005). Adiponectin: Action, Regulation and Association to Insulin Sensitivity. Obes. Rev..

[43] Polito R., Di Meo I., Barbieri M., Daniele A., Paolisso G., Rizzo M.R. (2020). Adiponectin Role in Neurodegenerative Diseases: Focus on Nutrition Review. Int. J. Mol. Sci..

[44] Haugen F., Drevon C.A. (2007). Activation of Nuclear Factor-ΚB by High Molecular Weight and Globular Adiponectin. Endocrinology.

[45] Beitz J.M. (2014). Parkinson s Disease a Review. Front. Biosci..

[46] Bloemer J., Pinky P.D., Govindarajulu M., Hong H., Judd R., Amin R.H., Moore T., Dhanasekaran M., Reed M.N., Suppiramaniam V. (2018). Role of Adiponectin in Central Nervous System Disorders. Neural Plast..

[47] Santaella A., Kuiperij H.B., van Rumund A., Esselink R.A.J., van Gool A.J., Bloem B.R., Verbeek M.M. (2020). Inflammation Biomarker Discovery in Parkinson’s Disease and Atypical Parkinsonisms. BMC Neurol..

[48] Kim J.-Y., van de Wall E., Laplante M., Azzara A., Trujillo M.E., Hofmann S.M., Schraw T., Durand J.L., Li H., Li G. (2007). Obesity-Associated Improvements in Metabolic Profile through Expansion of Adipose Tissue. J. Clin. Investig..

[49] Chinta S.J., Lieu C.A., DeMaria M., Laberge R.-M., Campisi J., Andersen J.K. (2013). Environmental Stress, Ageing and Glial Cell Senescence: A Novel Mechanistic Link to Parkinson’s Disease?. J. Intern. Med..

[50] Hoehn M.M., Yahr M.D. (1967). Parkinsonism: Onset, Progression, and Mortality. Neurology.

[51] Mańka S., Baj Z., Majewska E. (2019). The Influence of Melatonin on Apoptosis of Human Neutrophils. Postep. Hig. Med. Dosw..

[52] Cipolla-Neto J., Amaral F.G.D. (2018). Melatonin as a Hormone: New Physiological and Clinical Insights. Endocr. Rev..

[53] Vural E.M.S., van Munster B.C., de Rooij S.E. (2014). Optimal Dosages for Melatonin Supplementation Therapy in Older Adults: A Systematic Review of Current Literature. Drugs Aging.

[54] del Valle Bessone C., Fajreldines H.D., de Barboza G.E.D., Tolosa de Talamoni N.G., Allemandi D.A., Carpentieri A.R., Quinteros D.A. (2019). Protective Role of Melatonin on Retinal Ganglionar Cell: In Vitro an in Vivo Evidences. Life Sci..

[55] Cardinali D.P. (2019). Melatonin: Clinical Perspectives in Neurodegeneration. Front. Endocrinol..

[56] Grivas T.B., Savvidou O.D. (2007). Melatonin the “Light of Night” in Human Biology and Adolescent Idiopathic Scoliosis. Scoliosis.

[57] Scholtens R.M., van Munster B.C., van Kempen M.F., de Rooij S.E.J.A. (2016). Physiological Melatonin Levels in Healthy Older People: A Systematic Review. J. Psychosom. Res..

[58] Xie S., Fan W., He H., Huang F. (2020). Role of Melatonin in the Regulation of Pain. J. Pain Res..

[59] Tchekalarova J., Tzoneva R. (2023). Oxidative Stress and Aging as Risk Factors for Alzheimer’s Disease and Parkinson’s Disease: The Role of the Antioxidant Melatonin. Int. J. Mol. Sci..

[60] Breen D.P., Nombela C., Vuono R., Jones P.S., Fisher K., Burn D.J., Brooks D.J., Reddy A.B., Rowe J.B., Barker R.A. (2016). Hypothalamic Volume Loss Is Associated with Reduced Melatonin Output in Parkinson’s Disease. Mov. Disord..

[61] Gunata M., Parlakpinar H., Acet H.A. (2020). Melatonin: A Review of Its Potential Functions and Effects on Neurological Diseases. Rev. Neurol..

[62] Lin L., Du Y., Yuan S., Shen J., Lin X., Zheng Z. (2014). Serum Melatonin Is an Alternative Index of Parkinson’s Disease Severity. Brain Res..

[63] Videnovic A., Golombek D. (2017). Circadian Dysregulation in Parkinson’s Disease. Neurobiol. Sleep Circadian Rhythm..

[64] Kataoka H., Saeki K., Kurumatani N., Sugie K., Obayashi K. (2020). Melatonin Secretion in Patients with Parkinson’s Disease Receiving Different-Dose Levodopa Therapy. Sleep Med..

[65] Bolitho S.J., Naismith S.L., Rajaratnam S.M.W., Grunstein R.R., Hodges J.R., Terpening Z., Rogers N., Lewis S.J.G. (2014). Disturbances in Melatonin Secretion and Circadian Sleep–Wake Regulation in Parkinson Disease. Sleep Med..

[66] Durakoglugil M., Irving A.J., Harvey J. (2005). Leptin Induces a Novel Form of NMDA Receptor-Dependent Long-Term Depression. J. Neurochem..

[67] Ozdilek B., Kenangil G. (2014). Serum Leptin Concentrations in Turkish Parkinson’s Disease Population. Parkinsons. Dis..

[68] Fiszer U., Michałowska M., Baranowska B., Wolińska-Witort E., Jeske W., Jethon M., Piaścik-Gromada M., Marcinowska-Suchowierska E. (2010). Leptin and Ghrelin Concentrations and Weight Loss in Parkinson’s Disease. Acta Neurol. Scand..

[69] Evidente V.G.H., Caviness J.N., Adler C.H., Gwinn-Hardy K.A., Pratley R.E. (2001). Serum Leptin Concentrations and Satiety in Parkinson’s Disease Patients with and without Weight Loss. Mov. Disord..

[70] Markaki E., Ellul J., Kefalopoulou Z., Trachani E., Theodoropoulou A., Kyriazopoulou V., Constantoyannis C. (2012). The Role of Ghrelin, Neuropeptide Y and Leptin Peptides in Weight Gain after Deep Brain Stimulation for Parkinson’s Disease. Stereotact. Funct. Neurosurg..

[71] Adams F., Boschmann M., Lobsien E., Kupsch A., Lipp A., Franke G., Leisse M.C., Janke J., Gottschalk S., Spranger J. (2008). Influences of Levodopa on Adipose Tissue and Skeletal Muscle Metabolism in Patients with Idiopathic Parkinson’s Disease. Eur. J. Clin. Pharmacol..

[72] Bachmann C.G., Trenkwalder C. (2006). Body Weight in Patients with Parkinson’s Disease. Mov. Disord..

[73] Jung H.S., Park K.-H., Cho Y.M., Chung S.S., Cho H.J., Cho S.Y., Kim S.J., Kim S.Y., Lee H.K., Park K.S. (2006). Resistin Is Secreted from Macrophages in Atheromas and Promotes Atherosclerosis. Cardiovasc. Res..

[74] McTernan P.G., Kusminski C.M., Kumar S. (2006). Resistin. Curr. Opin. Lipidol..

[75] Kusminski C.M., Mcternan P.G., Kumar S. (2005). Role of Resistin in Obesity, Insulin Resistance and Type II Diabetes. Clin. Sci..

[76] De Rui M., Inelmen E.M., Trevisan C., Pigozzo S., Manzato E., Sergi G. (2020). Parkinson’s Disease and the Non-Motor Symptoms: Hyposmia, Weight Loss, Osteosarcopenia. Aging Clin. Exp. Res..

[77] Jenner P. (2008). Molecular Mechanisms of L-DOPA-Induced Dyskinesia. Nat. Rev. Neurosci..

[78] Kataoka H., Sugie K. (2020). Serum Adiponectin Levels between Patients with Parkinson’s Disease and Those with PSP. Neurol. Sci..

[79] Sekiyama K., Waragai M., Akatsu H., Sugama S., Takenouchi T., Takamatsu Y., Fujita M., Sekigawa A., Rockenstein E., Inoue S. (2014). Disease Modifying Effect of Adiponectin in Model of α-synucleinopathies. Ann. Clin. Transl. Neurol..

[80] Sharma J.C., Bachmann C.G., Linazasoro G. (2010). Classifying Risk Factors for Dyskinesia in Parkinson’s Disease. Park. Relat. Disord..

[81] Sharma J.C., Macnamara L., Hasoon M., Vassallo M., Ross I. (2006). Cascade of Levodopa Dose and Weight-Related Dyskinesia in Parkinson’s Disease (LD–WD-PD Cascade). Park. Relat. Disord..

[82] Cassani E., Cancello R., Cavanna F., Maestrini S., Di Blasio A.M., Liuzzi A., Pezzoli G., Barichella M. (2011). Serum Adiponectin Levels in Advanced-Stage Parkinson’s Disease Patients. Park. Dis..

